# Identification and characterization of a novel plaque-invisible lytic single-stranded RNA phage

**DOI:** 10.1128/jvi.01637-24

**Published:** 2025-11-10

**Authors:** Yuer Wang, Fengjuan Tian, Jinbei Zhang, Shan Xu, Mengzhe Li, Yigang Tong

**Affiliations:** 1 State Key Laboratory of Green Biomanufacturing, College of Life Science and Technology, Beijing University of Chemical Technology 47832, Beijing, China; St Jude Children's Research Hospital, Memphis, Tennessee, USA

**Keywords:** RNA phage, metagenomic, RT-qPCR, identification

## Abstract

The RNA phages offer promising applications in biotechnology, including vaccine development and drug delivery. However, their potential remains underexplored due to the limited number of known RNA phages, partly because conventional methods fail to identify plaque-invisible lytic phages that do not form plaques. Here, we introduced a novel method that combines RNA-inclusive metagenomic studies and quantitative reverse transcription-PCR (RMS-RT-qPCR) to identify and characterize active RNA phages from environmental samples. This study led to the discovery of a new active Qbeta-like phage, named Cute. Genomic analysis revealed that Cute is a new member of the *Qubevirus* genus. Although Cute does not form plaques, it can be observed to continuously release into the supernatant when co-cultured with the host by RT-qPCR detection. This discovery underscores the potential diversity of RNA phages in nature and the limitations of traditional culture-dependent techniques. Our findings suggest that RMS-RT-qPCR could aid in the discovery of active RNA phages with significant biotechnological applications.

**IMPORTANCE:**

The discovery and characterization of RNA phages might be historically constrained by traditional culture-based methods. Our study provides a powerful tool for identifying active RNA phages by combining RNA-inclusive metagenomic analysis with RT-qPCR. This method expands our understanding of the diversity and ecological roles of RNA phages, which are often overlooked in microbiome studies. This research highlights the importance of RNA phages in natural ecosystems and their potential applications in biotechnology and medicine, such as antimicrobial therapies and vaccine development. By expanding our understanding of RNA phage diversity, this study opens new avenues for their utilization in various fields, emphasizing the need for continued exploration of these versatile biological entities.

## INTRODUCTION

RNA phages offer promising applications in biotechnology, including vaccine development and drug delivery (1–10). However, the scarcity of RNA phages is one of the major factors limiting their in-depth study. As of 19 March 2024, the National Center for Biotechnology Information (NCBI) GenBank Genome database contains only seven genomic sequences of double-stranded RNA (dsRNA) phages, which belong to the *Cystovirus* genus of the *Cystoviridae* family. In addition, there are 883 genomic sequences of single-stranded (ssRNA) phages belonging to six families and 420 genera within the *Fiersviridae* family. Notably, no negative-strand RNA phage has been identified to date (11). In contrast, DNA phages are more prevalent in the environment, which are currently categorized into eight separate families by the International Committee on Taxonomy of Viruses (ICTV), and there are tens of thousands of genome sequences of DNA phage species in GenBank. By comparison, the number of characterized ssRNA phages is much smaller, which may reflect not only their biological features but also methodological and other limiting factors in their discovery.

The double-layer plate method is the conventional method for identifying phages (12). However, it is ineffective for detecting plaque-invisible lytic RNA phages, as some do not form plaques on agar plates. The traditional double-layer plate method may hinder the wide discovery of RNA phages. It is necessary to supplement or replace the traditional methods with other approaches. Although some studies have isolated the RNA phage phiNY that does not form plaque through CF11 cellulose (13), CF11 cellulose is only suitable for extracting dsRNA (only dsRNA can bind to cellulose) (14). Therefore, a more universal method for identifying RNA phages should be developed.

Metagenome sequencing based on next-generation sequencing (NGS) technology has been extensively employed for detecting pathogens (15) and drug-resistant genes (16). This technology is particularly advantageous for identifying novel viruses, significantly expanding the known viral repertoire (17–19). In 2016, Krishnamurthy et al. identified more than 120 novel RNA phages by analyzing existing metagenomic databases, which previously contained only 11 ssRNA and five dsRNA phage genome sequences in the NCBI (20). In 2020, Wolf et al. identified more than 4,500 different RNA viruses based on metagenomic analysis of water samples (21). In addition, Callanan et al. identified complete genome sequences of more than 1,000 novel ssRNA phages in publicly available metatranscriptomic databases (11). These studies demonstrated that only a limited portion of RNA phages present in the environment has been characterized. These studies have significantly enriched the known diversity of RNA phages, uncovering several structural domains that had not been previously reported in these phages, thereby facilitating evolutionary analysis and the exploration of gene structure-function relationships. However, the analysis is limited to the genetic level due to the lack of active phages (22). Obtaining active phages is necessary for a more in-depth investigation of their evolutionary relationships with hosts and for exploring their practical applications.

In our study, we combined RNA-inclusive metagenomic studies with quantitative reverse transcription-PCR (RMS-RT-qPCR) to identify and culture a novel RNA phage, determine its host, and monitor its lifecycle through RT-qPCR.

## RESULTS AND DISCUSSION

### Metagenomic analysis and identification of ssRNA phage sequence

**Fig 1:**
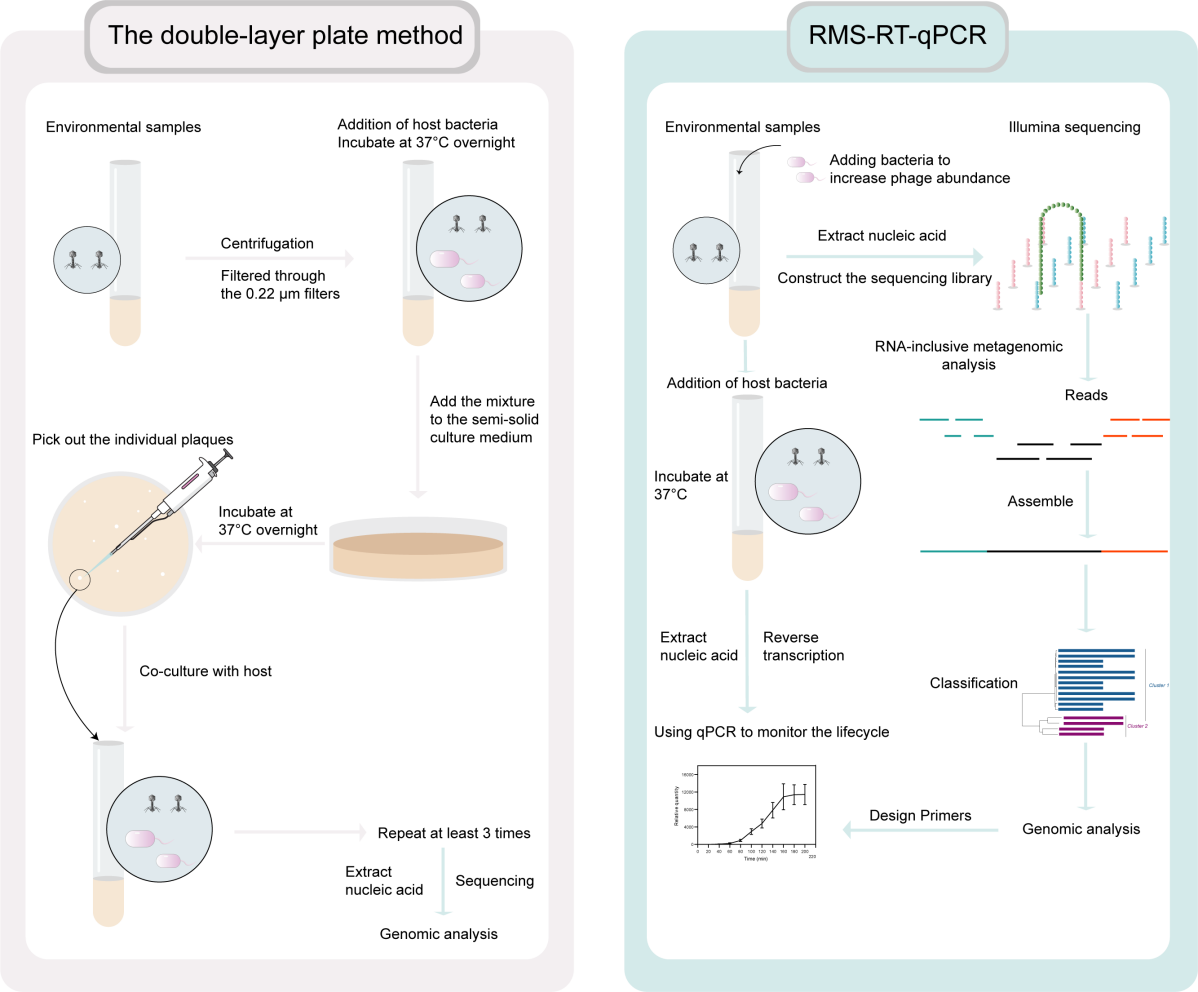
Flowchart for the discovery of novel phages in the environment based on the double-layer plate method and RMS-RT-qPCR approach.

Using the RMS-RT-qPCR method (Fig. 1), we identified a novel lytic ssRNA phage, named Cute, from the metagenomic data of the soil sample. Since it is challenging to identify RNA phages by analyzing metagenomic data directly from environmental samples due to their low abundance (11), we addressed this issue by increasing phage abundance within samples through adding pure cultures of different bacteria and incubating them overnight before constructing the metagenomic library.

**Fig 2:**
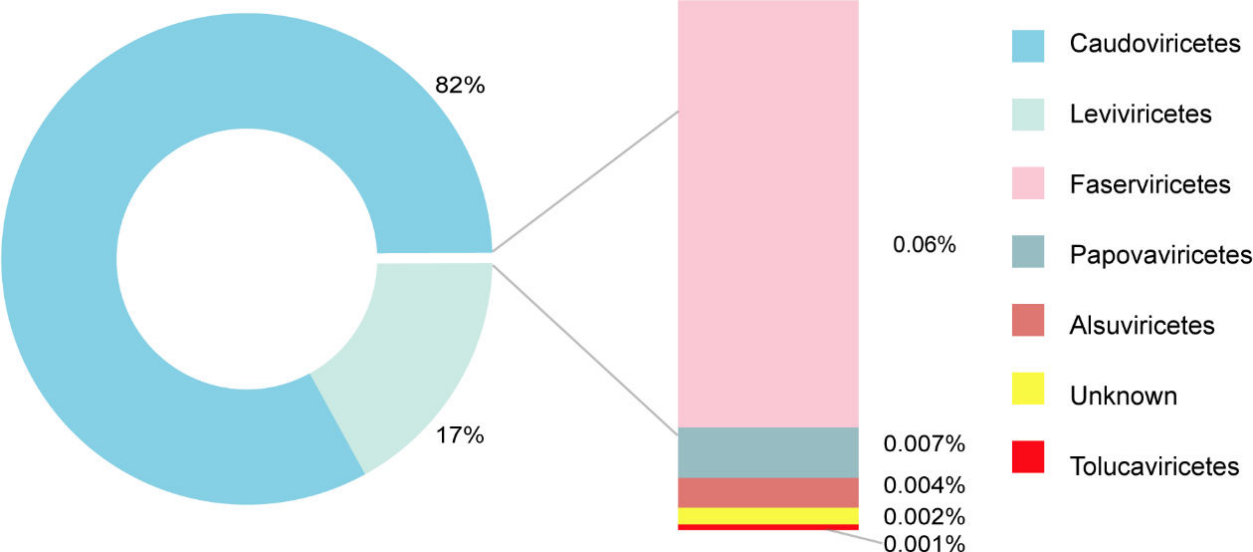
Phage composition of the sample. Phage classes are color-coded. The percent values for each phage group are shown.

We initially processed raw sequencing data from the metagenomic library constructed on the Illumina platform, yielding a total of 70,391,221 reads with an average length of 150 bp and an average GC content of 62%. After quality control and assembly using SPAdes, we identified 473 phage contigs using VirSorter2 and checkV for viral sequence identification. Of these, 389 contigs were classified into seven different viral classes. We calculated the transcripts per million for each contig, summed them by category, and then calculated the relative abundance ratios for each classification: *Caudoviricetes* (82.91%), *Leviviricetes* (17.01%), *Faserviricetes* (0.06%), *Papovaviricetes* (0.004%), *Alsuviricetes* (0.002%), and *Tolucaviricetes* (0.001%) (Fig. 2). Notably, the significant presence of *Leviviricetes* suggests a potential abundance of ssRNA phages in this sample. To further verify our hypothesis and focus on RNA viruses, we reanalyzed the sequences using Virsorter2 for RNA virus detection and aligned the reads against the NCBI non-redundant nt database using BLASTn. This reanalysis identified a sequence with 74.91% homology (Query Cover 79%) with *Enterobacteria* phage Qbeta (NC_001890.1) and 95.49% homology (Query Cover 99%) with *Escherichia* phage Qbeta GIII_C RNA (LC710219.1). Based on this high degree of sequence similarity, we hypothesize the presence of a novel ssRNA phage in our metagenomic data set.

### Genomic analysis of phage Cute

**Fig 3:**
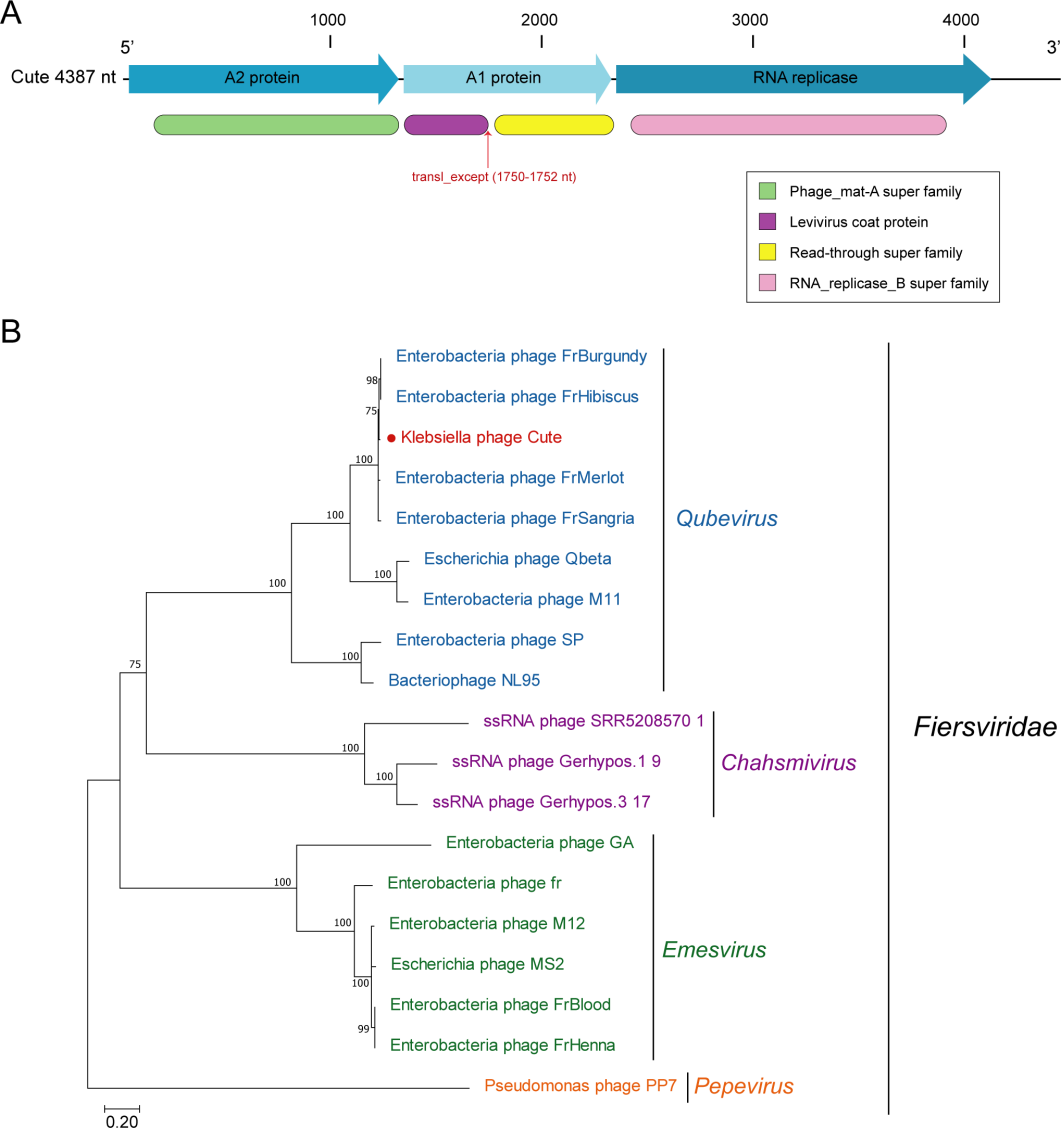
Schematic representation of the genome structure of phage Cute and phylogenetic analysis of Cute. (**A**) The genome is 4,387 nt long and contains three long possible ORFs and four conserved structural domains. The three ORFs encode A2 maturation protein (nt 68–1330), A1 minor capsid protein (nt 1351–2349), and RNA replicase (nt 2368–4137). (**B**) The amino acid sequences of RNA replicase were aligned by MAFFT. The maximum likelihood (ML) tree was constructed by Figtree. The optimal model predicted by IQ-TREE was LG+G+I. Bootstrap values were obtained from 1,000 replicates. Branch scale bars are shown as 0.20 substitutions per site. Different genera of viruses in the family *Fiersviridae* are distinguished by different colors. The red color denotes the novel phage, Cute.

In our analysis of the novel phage, named Cute, we identified a genome spanning 4,387 nt with a G+C content of 48%. The genome architecture of Cute is linear, containing three open reading frames (ORFs) and four conserved structural domains. Specifically, the ORFs encode the A2 maturation protein, the A1 minor capsid protein, and the RNA replicase (Fig. 3A). The conserved domains are characterized as the Phage_mat-A superfamily, the Levivirus coat protein, the Read-through superfamily, and the RNA_replicase_B superfamily. Phylogenetic analysis based on RNA replicase amino acid (aa) sequences positions the phage Cute within the genus *Qubevirus* in the family *Fiersviridae* (Fig. 3B). The phage Cute clustered in a well-supported clade, including the *Escherichia* phage Qbeta (GenBank accession no. NC_001890.1 ) and the *Escherichia* phage FrHibiscus (GenBank accession no. PP430143.1). The translational exception of the phage Cute at nucleotides 1750–1752, where the stop codon TGA is occasionally bypassed, allows for continued translation. As previously reported, this mechanism results in the production of the A1 minor capsid protein, albeit in low quantities (about 3–10 copies per virus particle) (23). This discovery of Cute expands our understanding of RNA phage genome organization and highlights the diversity within the *Fiersviridae* family, offering new insights into the genetic and functional complexities of ssRNA phages.

### Morphological characterization and biological characteristics of phage Cute

**Fig 4:**
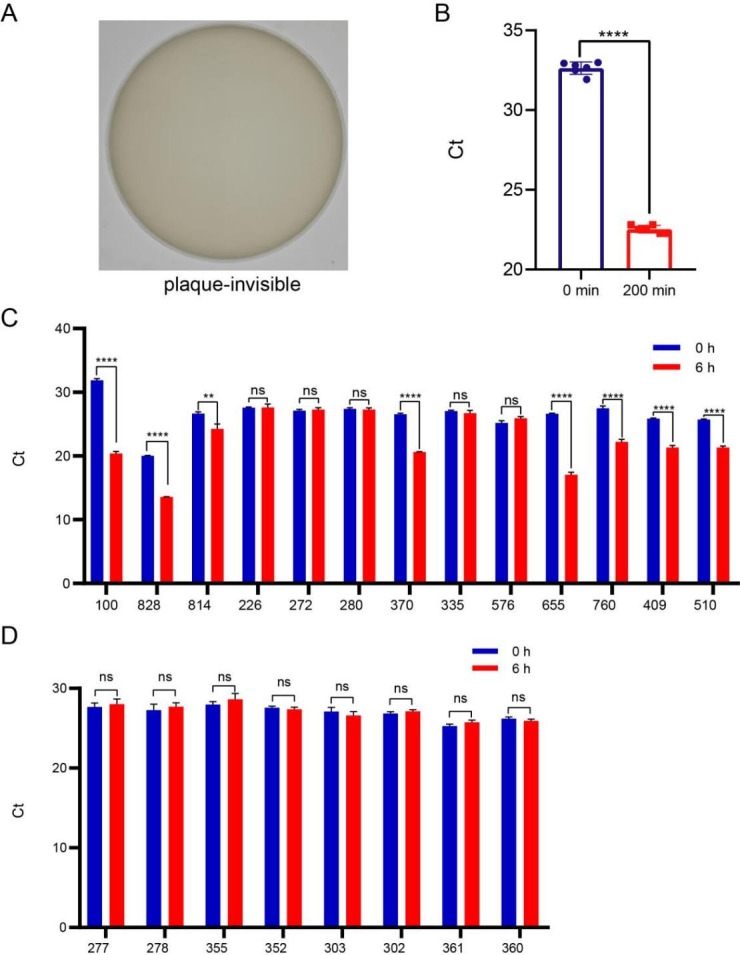
Identification of phage Cute hosts. (**A**) The double-layer agar plate assay shows that the phage Cute did not form any plaque on the host *K. pulmonicu*s 100. (**B**) The RT-qPCR was used to ascertain their host range. The specific primers of phage Cute *A1* gene were used to detect the Ct values of phage Cute at the initial time (blue data, 0 min) and in the supernatant of phage Cute co-cultured with the host *K. pulmonicu*s 100, 37°C for 200 min (red data, 200 min) by RT-qPCR. (**C**) The results of RT-qPCR showed that several *K. pneumoniae* strains (e.g., 100, 370, 409, 510, 655, 760, 814, and 828) supported phage replication, as evidenced by markedly reduced Ct values after co-culture. (**D**) The RT-qPCR results showed that no changes in Ct values were observed after co-culture of phage Cute with *Escherichia coli* strains. Significant differences between groups linked by horizontal lines are indicated by asterisks: ns indicates no significant difference; **P < 0.01, ****P < 0.0001.

**Fig 5:**
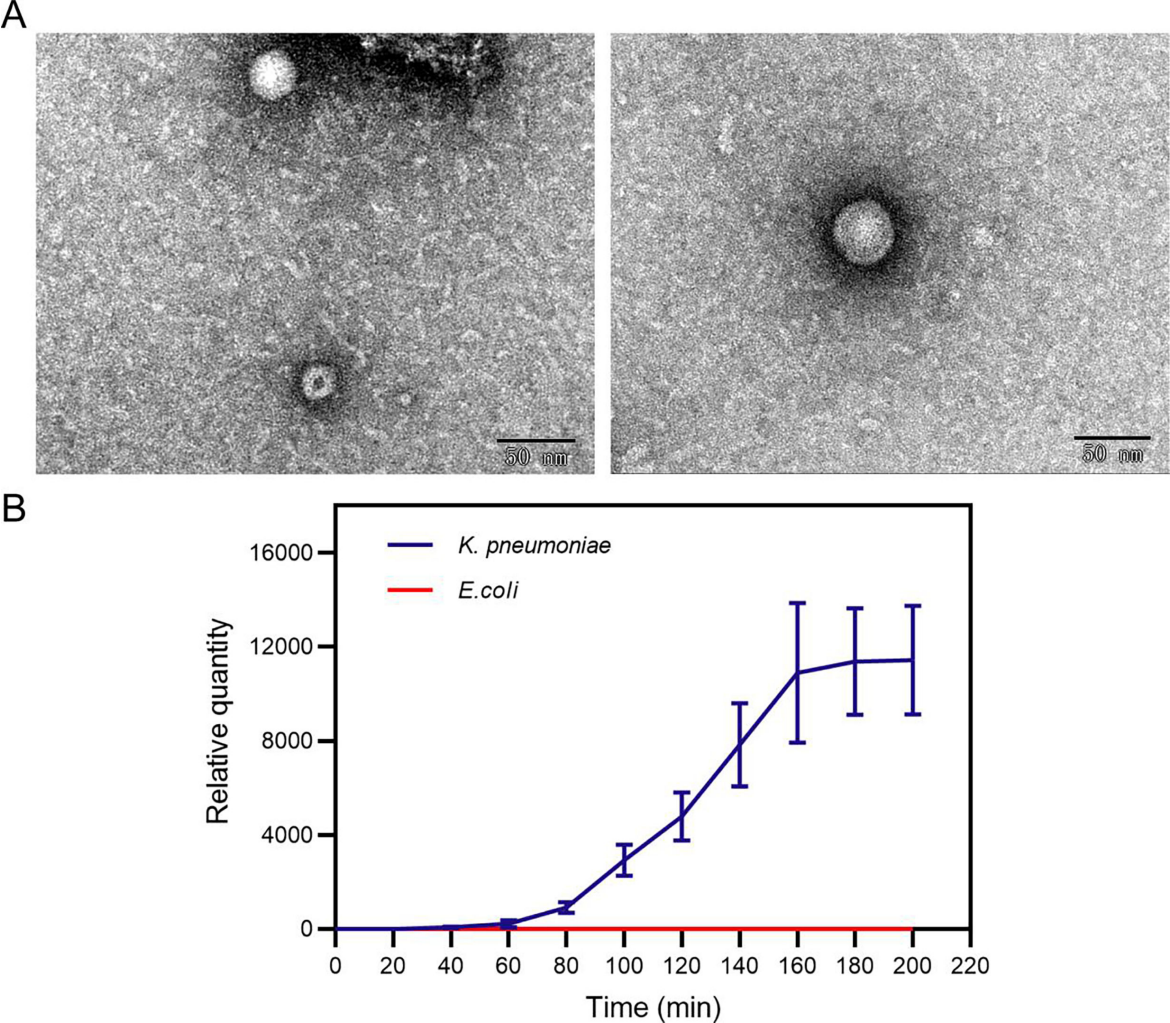
Morphology of the phage Cute revealed by transmission electron microscopy. (**A**) The phage Cute has an icosahedral structure with a diameter of about 28 nm. (**B**) Relative quantity curve with the RT-qPCR method. The $\;2^{\Delta ct}$ method was used to draw the relative quantity curve of the phage Cute by GraphPad Prism 9.

We used the specific primer of phage Cute *A1* gene to detect the Ct value of phage Cute at the initial time (blue column, 0 min) and in the supernatant after co-culturing with the original host *Klebsiella pneumoniae* (*K. pneumoniae*) 100, 37°C for 200 min (red column, 200 min) by RT-qPCR method. The results showed that the Ct value decreased significantly after co-culture with the original host *K. pneumoniae* 100 (Fig. 4B), indicating that although the phage Cute did not form plaques (Fig. 4A), it could be released into the supernatant after co-culture with the host. Transmission electron microscopy (TEM) shows that the phage Cute is a tailless phage with a diameter of approximately 28 nm (Fig. 5A), which resembles the morphology of the previously described (1). After negative staining, phage Cute appears as uniformly sized bright round particles. Due to its icosahedron structure, it may present a hexagonal appearance at certain angles. The growth experiment showed a latent period of 60 min and a burst period of 100 min, followed by the plateau phase after 160 min (Fig. 5B).

### Host range analysis

Since phage Cute does not form any plaques on these strains, we, respectively, used the machine learning software vpf-class (24, 25) and the CRISPR-based analysis method (26, 27) to predict the hosts of phage Cute, but both failed. Predicting RNA phages using bioinformatics remains a challenge (28, 29). To overcome these limitations, we employed RT-qPCR to ascertain the host of phage Cute (30). We co-cultured the phage Cute with 13 strains of *K. pneumoniae* and 8 strains of *Escherichia coli* (*E. coli*) and subsequently performed RT-qPCR targeting the *A1* gene of phage Cute. The results showed that several *K. pneumoniae* strains (e.g., 100, 370, 409, 510, 655, 760, 814, and 828) supported phage replication, as evidenced by Ct values after co-culture markedly reduced (Fig. 4C). In contrast, no comparable changes were observed with *E. coli* strains (Fig. 4D). Taken together, these results corroborate that *K. pneumoniae* serves as the host of phage Cute, with 8 out of 13 strains being hosts of phage Cute. Given that it has been shown that most *Qubevirus* phages with *E. coli* as the host, this would be the first evidence that the host range of *Qubevirus* phages has been extended to include *K. pneumoniae*.

### The TraA protein serves as the receptor of the phage Cute

**Fig 6:**
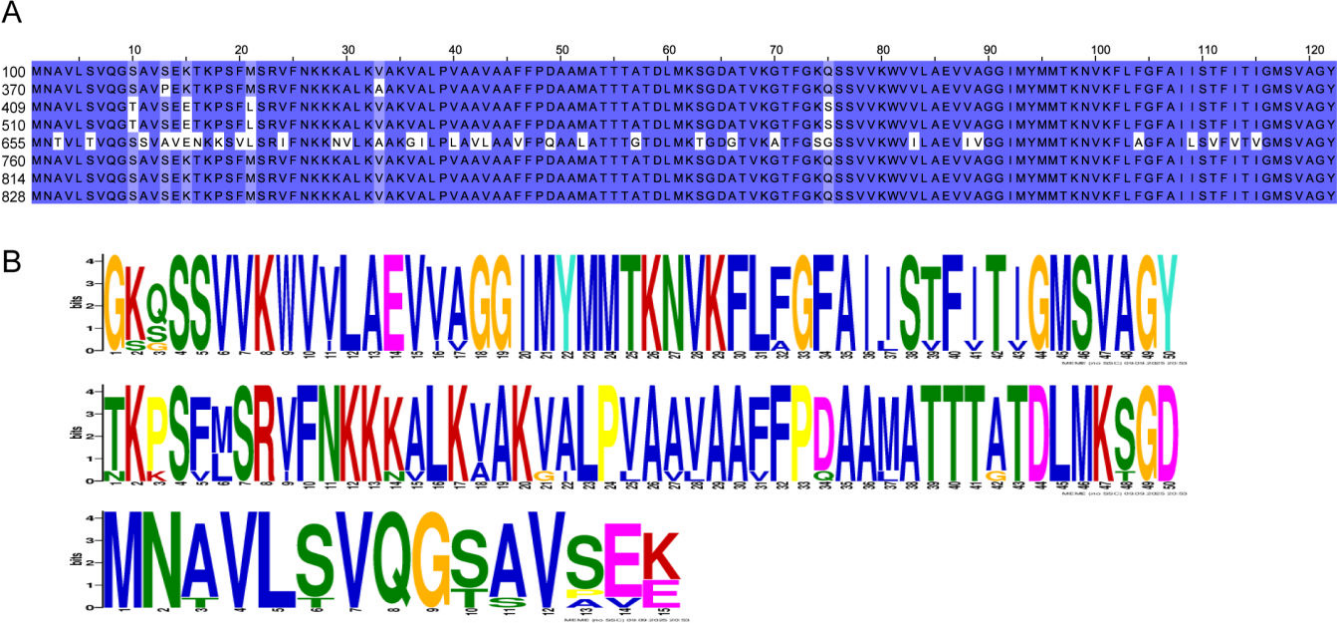
(**A**) Alignment of aa sequences of the TraA protein from 13 *K*. *pneumoniae* strains (e.g., 100, 370, 409, 510, 655, 760, 814, and 828). (**B**) Three conserved motifs in the aa sequence of the TraA protein.

Previous studies have shown that F-pilus is the attachment site of RNA phages R17 and Qbeta (31), and the A2 protein of the phage Qbeta is responsible for adsorption on F-pilus (2) (32, 33). The F-pilus is composed of F-pilin. The F-pilin subunit is processed from a 121 aa propilin encoded by the *traA* gene. Previous studies have shown that four random mutations in the *traA* gene can affect the attachment sites of RNA phages on F-pilus (34). To investigate whether the infection of *K. pneumoniae* by phage Cute might be associated with the F-like conjugation plasmid pKpQIL, we sequenced the genomes of 13 *K*. *pneumoniae* strains. The results showed that only the original host *K. pneumoniae* 100 and seven other susceptible bacteria (i.e., strains that can be adsorbed and infected by phage Cute) have the F-like conjugation plasmid pKpQIL, which carries the *traA* gene. This observation suggests a potential relationship between the presence of TraA and phage Cute susceptibility. The aa sequences of the TraA protein of 13 strains of *K. pneumoniae* were aligned, and it was found that these sequences were highly similar (Fig. 6A). We discovered three conserved aa motifs from it, which may determine the attachment of the A2 protein of phage Cute (Fig. 6B).

Although the development of cryo-electron microscopy has contributed to the study of the interaction mechanism of ssRNA phages binding to their receptors (35), there are fewer studies on the specific molecular mechanism of the interaction between the A2 protein and TraA. Since the A2 maturation/lysis protein of phage Qbeta is responsible for adsorption and lysis, changes in the host range of the phage Cute may be related to alterations in the aa sequence of the A2 protein. Initially, we aligned the nucleotide and aa similarities between phage Cute A2 and Qbeta A2, which are 72.79% and 63.90%, respectively. To further explore the reasons, we aligned the aa sequence of the Cute A2 protein with other *Enterobacteria* phages in *Qubevirus* with *E. coli* as host (Fig. S1). The aa highlighted in red represents non-conserved aa residues in the phage Cute A2 sequence. We speculate that the changes in these aa positions are the key factors in the variation of the phage host range. However, the specific mechanism still requires further investigation.

### Exploring the potential application value of the phage Cute

The virus-like particles (VLPs) based on the phage Cute have a broad perspective in applications, such as bioimaging, drug delivery, and vaccines (36), due to their ability to self-assemble *in vitro* and display antigenic epitopes on the coat protein surface (37, 38). The formation of functional VLPs depends on the specific binding of coat protein to the RNA operator. Therefore, predicting the RNA operators in the genome of phage Cute is of crucial importance for exploring the application potential of ssRNA phages.

**Fig 7:**
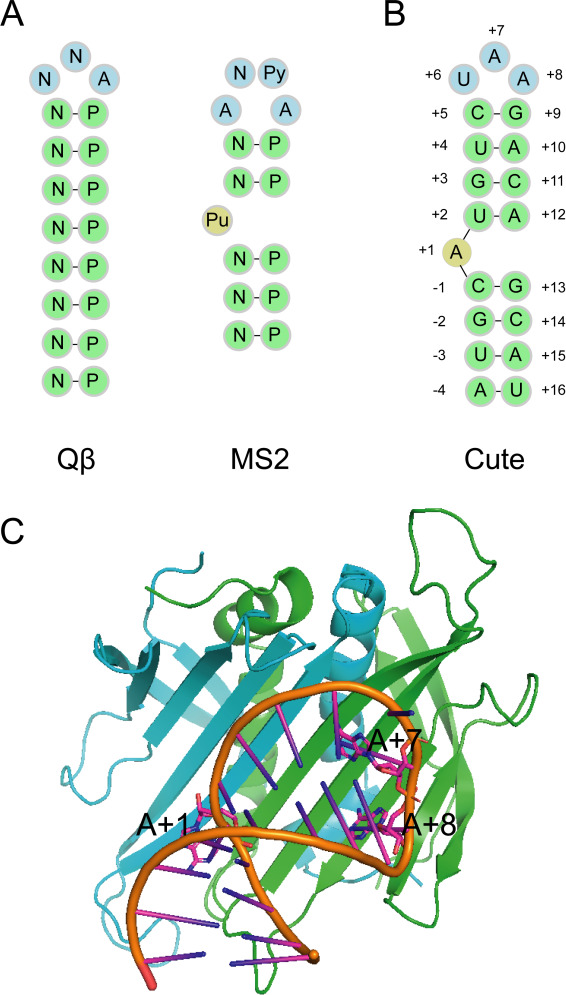
The secondary structure of the Cute RNA operator sequence and the molecular docking between the RNA operator and the Cute CP. (**A**) The RNA operators’ basic structures of the phage Qβ and MS2. The RNA operators of the phage Qβ and MS2 consist of an adenine-containing loop and a stem, which differ in the size of the loop and the length of the stem. Py denotes pyrimidine; Pu denotes purine; N denotes any nucleotide; P denotes nucleotide complementary to N. (**B**) The phage Cute RNA operator (AUGCAUGUCUAAGACAGCAU) is located at 2,364–2,383 nt in the genome. (**C**) Three-dimensional structure of the phage Cute coat protein-operator complex. The coat protein dimer is represented in green (monomer A) and blue (monomer B).

The phage Qbeta has a similar structure in the RNA operators to the phage MS2 (Fig. 7A) (39). The RNA operators of both Qbeta and MS2 consist of an adenine-containing loop and a stem, which differ in the size of the loop and the length of the stem. The coat protein of MS2 recognizes the Qbeta RNA operator by replacing the glutamic acid in residue 89 with threonine or lysine (40). The key structure of the Qbeta RNA operator is a three-nucleotide loop and an eight-base-pair stem, with the last adenine in the loop necessary to occupy the adenine-binding pockets of the CP. Notably, we found that phage Cute shares the same RNA operator sequence (AUGCAUGUCUAAGACAGCAU) with phage Qbeta (Fig. 7B), which is located between 2,364 and 2,383 nt in the genome of phage Cute.

**Fig 8:**
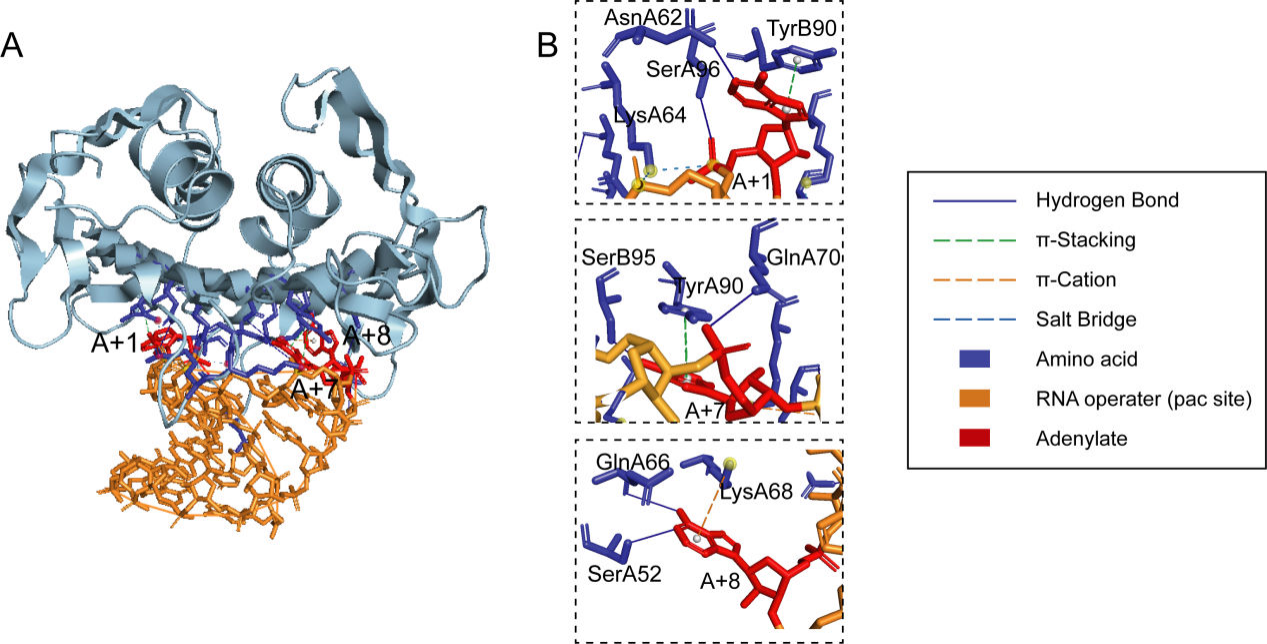
(**A**) Schematic diagram of phage Cute binding to RNA operator. Gray represents coat protein dimers, yellow represents RNA, red represents adenylate, and blue represents aa residues that interact with adenine. (**B**) Close-up view of protein-RNA interactions in phage Cute. The figure shows the recognition pocket structures of A+1, A+7, and A+8, where adenylate (red) and aa residues (blue) are presented in a rod-shaped model.

To confirm the accuracy of the phage Cute RNA operator sequence, we simulated the molecular docking between the three-dimensional structure of the phage Cute coat protein dimer and the RNA operator with HDOCK software (Fig. 7C). The results demonstrate that the binding site between the coat protein dimer and the RNA operator is located on the surface of the β-sheet, which is consistent with previous research findings (39). As reported previously, A+1 and A+7 bases are stacked together with the Tyr of the two monomers in the model of coat protein dimer and RNA operator, respectively (Fig. 8A and B) (41). The adenine base of A+8 nucleotides is suitable for the adenine binding bag formed by Ser, Gln, and Lys of the A chain in the capsid protein dimer (Fig. 8B). In summary, our predicted RNA operator demonstrates a strong ability to bind within the protein pocket, confirming the reliability of the predicted RNA operator sequence.

To further confirm the accuracy of the RNA operator sequence of the phage Cute, we prepared Cute VLP using prokaryotic expression vectors (Fig. S2A). In addition, enhanced green fluorescent protein (EGFP) carrying RNA operator was used as the target sequence, and RT-qPCR was used to verify whether the Cute VLP was packaged with cargo RNA after extracting nucleic acid. Naked RNA samples extracted from Cute VLPs were treated without reverse transcriptase (the reverse transcriptase was replaced with RNase-free water) as a control (42). Compared with the unreverse transcription samples (non-RT), the Ct value of the reverse transcription (RT) was significantly decreased, which demonstrated that the target RNA EGFP was indeed encapsulated in Cute VLP (Fig. S2B).

In conclusion, we successfully developed an innovative approach for identifying and characterizing plaque-invisible lytic phage. Our method, RMS-RT-qPCR, combines RNA-inclusive metagenomic studies with RT-qPCR analysis, which not only facilitates the discovery of novel RNA phage genomes but also enables the identification of active phage. Utilizing this approach, we identified a new Qbeta-like phage from soil samples, and it belonged to the *Qubevirus* genus in the *Fiersviridae* family. Host range assays revealed that it could infect *K. pneumoniae*. Our findings can expand the known diversity of RNA phages and emphasize their potential ubiquity in the environment. This study paves the way for further exploration of RNA phage ecology and evolution, and discovered more RNA phages as candidate vehicles for vaccine delivery, gene therapy, and antimicrobial treatments.

## MATERIALS AND METHODS

### Sample collection and preprocessing

Soil samples were collected from an area near the 307 Hospital of the People’s Liberation Army in Beijing, China. The 100 g of soil samples were then resuspended in 500 mL of phosphate-buffered saline (PBS) and centrifuged at 10,000 × *g* for 10 min. Next, 115 bacterial strains representing 18 different species (Table S1) in the Tong Lab bacterial library were selected as candidate hosts for RNA phages. The bacteria were, respectively, cultured in 10 mL shaking tubes to the logarithmic phase. Specifically, we added 50 µL of soil suspension per tube to 115 shaking tubes, each containing 5 mL of bacterial culture in the logarithmic phase. After overnight incubation, 50 µL from each culture was transferred to a clean centrifuge tube, resulting in a total of 5,750 µL of enriched mixture. Then, 200 µL of the enriched mixture was used to extract nucleic acids, and one library was constructed for subsequent NGS analysis.

### Next-generation sequencing

The co-cultures were centrifuged at 10,000 × *g* for 10 min, after which the supernatant was filtered through 0.22 µm filters to remove bacteria. Viral RNA was extracted from the filtrate using the High Pure viral RNA Kit (Roche, Switzerland) according to the manufacturer’s instructions. A paired-end library was then prepared with the NEBNext Ultra II Directional RNA Library Prep Kit for Illumina (NEB, Germany). The quality of the constructed library was assessed using RT-qPCR (Applied Biosystems, Thermo Fisher Scientific, USA). Finally, the prepared libraries were sequenced using NovaSeq S2 reagent kits on a NovaSeq 6000 platform (Illumina, USA).

### Metagenomic analysis and RNA phage discovery

Fastp (v0.20.0) and BBNorm were applied for quality control and repetition of sequencing reads. The resulting reads were aligned against viral genome databases using Diamond blastx (v0.9.7) to identify virus-associated reads. Then, viral reads were compared against the NCBI non-redundant nucleotide (nt) database by BLASTn, and taxonomic lineage information was derived from the top BLAST hit of each read. Next, the reads were assembled into contigs using metaSpades (v3.15.5) with default settings, keeping contigs with a minimum length of 1.5 kb for further analysis. Afterward, Virsorter2 (v2.2.3) was employed with a loose cut-off of 0.5 to maximize sensitivity in identifying contigs potentially containing viral sequences, acknowledging that some non-viral sequences or regions might also be included. Quality control of the VirSorter2 results was performed using CheckV, which was used to trim potential host regions at the ends of proviruses and discard sequences lacking viral genes. Redundant sequences were removed using cdhit (v4.8.1). The remaining sequences were classified using Genomad (v1.7.4). Read mapping to calculate abundance was performed as described in the section “Metagenomic analysis and identification of ssRNA phage sequence.”

### Bioinformatics analysis

Putative ORFs were predicted using the ORF finder tool available at NCBI (https://www.ncbi.nlm.nih.gov/orffinder/). Conserved structural domains within predicted ORFs were identified using the NCBI Conserved-Domain Search (https://www.ncbi.nlm.nih.gov/Structure/cdd/wrpsb.cgi). The secondary structures of RNA operators were predicted with RASP v2.0 (http://rasp2.zhanglab.net/) (43). The aa sequences of RNA replicase of Cute and other *Fiersviridae* viruses (Table S2) were aligned by MAFFT (44). Phylogenetic analysis of these aligned sequences was conducted with IQ-TREE (45), with the optimal model LG+G+I. The ML tree was constructed using Figtree (46), with bootstrap values (47) derived from 1,000 replicates and branch scale bars representing 0.20 substitutions per site. The three-dimensional structure of the phage Cute coat protein was modeled using SWISS-MODEL (48). Molecular docking and coat protein dimer predictions were performed with HDOCK software (49).

### Transmission electron microscopy

Phages were purified by sucrose density gradient centrifugation (30% sucrose, 35,000 × *g* for 2 h) as previously reported (50, 51). Specifically, the phage Cute was co-cultured with the host at 37°C and 200 rpm overnight. The culture medium was centrifuged at 10,000 × *g* for 5 min, and the supernatant was filtered through a 0.22 µm filter. Add 6 g of sucrose to 20 mL of filtrate to prepare a 30% wt/vol sucrose solution. Add the prepared sucrose buffer to a 38.5 mL Ultra-Clear centrifuge tube (Beckman Coulter). Centrifuge the tubes in a SW 32 Ti rotor (Beckman Coulter) at 35,000 × *g* for 2 h. Take the bottom solution and resuspend it with 200 µL PBS. The purified phage Cute solution was incubated on a carbon-coated copper grid for 10 min and then dried using filter paper. The sample was then negatively stained with uranyl acetate for 90 s and air-dried at room temperature. Phage morphology was subsequently observed by TEM (JEM-1400plus, Japan) at 120 kV (52).

### Growth curve experiment

Phage Cute was mixed with its host *K. pneumoniae* for 5 min, and the mixture was then centrifuged at 10,000 × *g* for 5 min to remove unadsorbed phages. The precipitate was resuspended with Luria-Bertani (LB) and cultured at 37°C with shaking at 200 rpm. The co-cultures were subsequently centrifuged at 10,000 × *g* for 10 min, and the supernatant was filtered through the 0.22 µm filters. RNA extraction, cDNA synthesis, and qPCR were performed as described in “Host range analysis,” below. The relative quantity curve of phage Cute was plotted using the $\;2^{\Delta }$^Ct^ method (53) by GraphPad Prism 9.

### Double-layer plate method

The double-layer plate method was experimentally performed as described previously (54). Briefly, the base layer of LB solid medium (1.5% agar) was first prepared. Then, 100 µL of the overnight cultured *K. pneumoniae* strain 100 and 100 µL of phage Cute (filtered through a 0.22 µm filter) were mixed with 5 mL of semi-solid LB medium (0.75% agar). This mixture was then overlaid onto the base layer and incubated overnight at 37°C.

### Host range analysis

The 50 µL of each bacterial strain and 50 µL of the phage Cute (through a 0.22 µM filter) were mixed and incubated for 5 min, followed by centrifugation at 10,000 × *g* for 5 min to remove unadsorbed phage. Resuspend the precipitate with 5 mL LB. At the initial moment, samples were taken and passed through the 0.22 µm filter. After co-culture at 37°C for 6 h, samples were taken again and passed through the 0.22 µm filter. Subsequently, the filtrate was lysed at 100°C for 5 min to release the RNA. HiScript II Q RT SuperMix for qPCR (+gDNA wiper) (Vazyme, China) was used to prepare cDNA. The qPCR primers were designed to detect the phage Cute *A1* gene (Table S3). The qPCR was performed using Taq Pro Universal SYBR qPCR Master Mix (Vazyme, China) on the 7500 real-time PCR system.

### Cute VLP plasmid construction and expression system

The DNA sequences of the phage Cute’s coat protein dimer with his tag, *A2* gene, and the sequences of EGFP were synthesized by General Biol Co., Ltd., and the target sequences were constructed into the PACYC plasmid by homologous recombination. The obtained plasmids were verified by Sanger sequencing (General Biol Co., Ltd., China) and named coatdimer-EGFP-pac. The coatdimer-EGFP-pac plasmid was transformed into the BL21(DE3) strain as a prokaryotic expression system. BL21(DE3)-coatdimer-EGFP-pac was cultured at 37°C in LB (400 mL) supplemented with 50 µg/mL chloramphenicol. The expression was induced by adding 160 µL 1 M isopropyl-beta-D-thiogalactopyranoside (IPTG) at OD 600 = 0.8 at 16°C and 200 rpm for 24 h. The bacteria were precipitated by centrifugation (7,000 rpm, 10 min, 4°C), resuspended in 10 mL PBS, and centrifuged at 6,000 rpm for 10 min (4°C). The following were added to the bacterial sediment: 20 mL of Tris-HCl NaCl buffer containing 500 µL of 30% Triton X-100, 6 µL of 100 mg/mL RNase (Vazyme Biotech Co., Ltd.), 600 µL of 1 mg/mL DNase I (Solarbio), 3 mL of glycerol, and 300 µL of protease inhibitor (Selleckchem). The bacterial precipitate suspension containing VLPs was disrupted by ultrasonic homogenizer and then centrifuged in the ultracentrifuge (SW32 Ti; 38.6 mL tube capacity) at 4°C, 18,000 rpm for 20 min. The supernatant containing his-tagged VLPs was purified by Ni-NTA Agarose (QIAGEN). To remove the plasmids from the solution, we pre-incubated VLP solution with 150 U of DNase I at 37°C for 1 h. Subsequently, DNase I was thermally inactivated at 68°C for 10 min. Then, the impurity proteins were eluted successively with Solution A (30 mM imidazole) and Solution B (60 mM imidazole). Finally, VLP was eluted with the eluents (500 mM imidazole), and the purified VLP was stored at 4°C.

## Data Availability

The GenBank accession number for phage Cute is OP321095.

## SUPPLEMENTAL MATERIAL

Supplemental material jvi.01637-24-s0001.docx Figures S1 and S2; Tables S1 to S3.

ASM does not own the copyrights to Supplemental Material that may be linked to, or accessed through, an article. The authors have granted ASM a non-exclusive, world-wide license to publish the Supplemental Material files. Please contact the corresponding author directly for reuse.
