# Supplementary material for: Identification and characterization of a novel plaque-invisible lytic single-stranded RNA phage
Source: J Virol. 2025 Nov 10;99(12):e01637-24. doi: 10.1128/jvi.01637-24 (PMC12724129; doi:10.1128/jvi.01637-24)
Supplement: Supplemental material — Figures S1 and S2; Tables S1 to S3. [file jvi.01637-24-s0001.docx]

**Supplemental Material**

**Identification and Characterization of a Novel Plaque-Invisible Lytic Single-Stranded RNA Phage**

Yuer Wang^1#^, Fengjuan Tian^1#^, Jinbei Zhang^1#^, Shan Xu^1*^, Mengzhe Li^1*^,Yigang Tong^1*^

^1^ State Key Laboratory of Green Biomanufacturing, College of Life Science and Technology, Beijing University of Chemical Technology, Beijing 100029

^#^These authors contributed equally to this work.

^*^Correspondence: Shan Xu: shanxu@buct.edu.cn; Mengzhe Li: futurelmz123@163.com; Yigang Tong: tongyigang@mail.buct.cn.

**
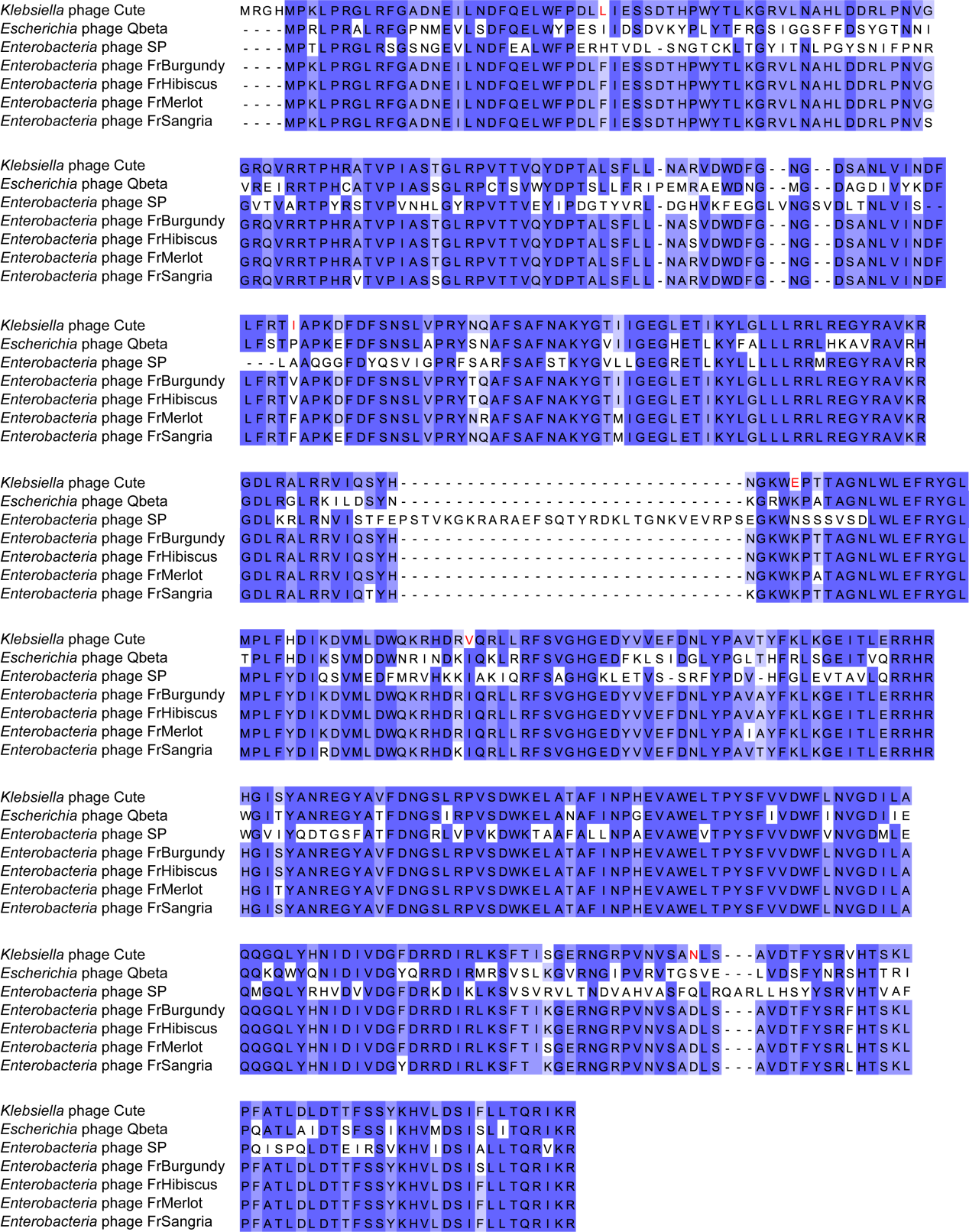
**

**Figure S1. The amino acid sequence of the Cute A2 protein was aligned with other *Enterobacteria* phages in *Qubevirus* with *E. coil* as host.** The shade of blue represents the degree of sequence conservation, the darker the color, the more conserved. The aa highlighted in red represents non-conserved aa residues in the phage Cute A2 sequence.

**
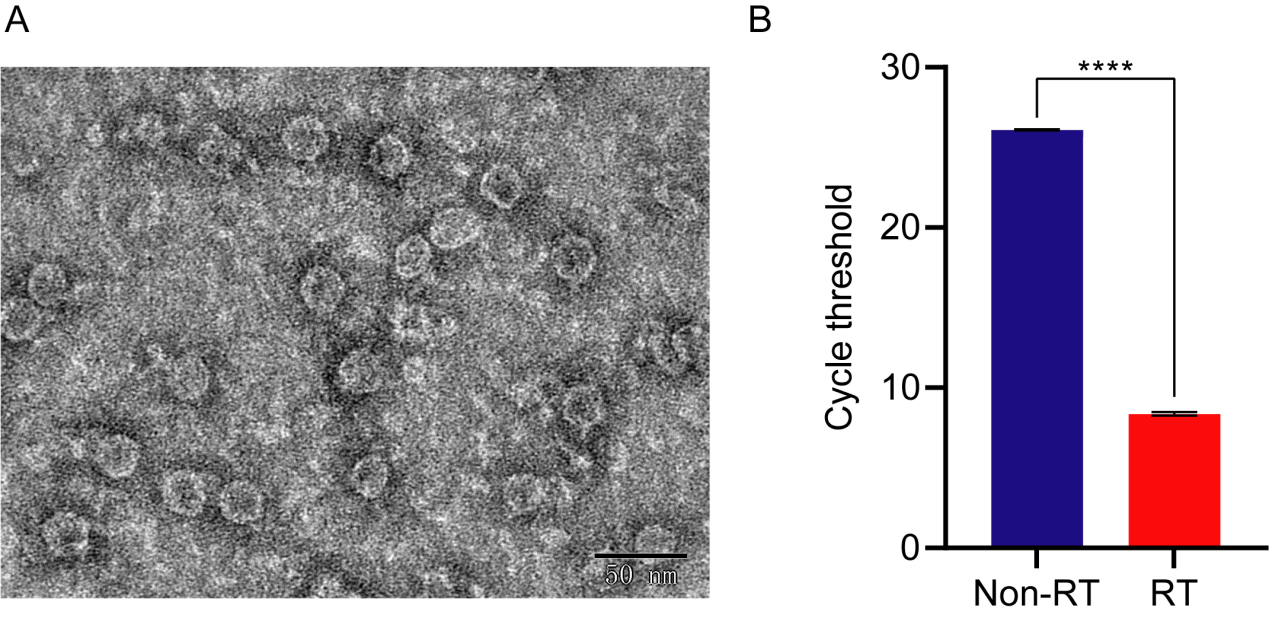
**

**Figure S2. (A) TEM image of Cute VLP prepared from prokaryotic expression vectors. (B) RT-qPCR was used to verify whether Cute VLP packaged RNA.** Naked RNA samples extracted from Cute VLPs were used as control without reverse transcriptase (Nuclease-free water was used instead of the reverse transcriptase). Compared with the unreverse transcription sample (Non-RT), the Ct value of the reverse transcription sample (RT) was significantly decreased, indicating that the target RNA EGFP was indeed encapsulated in Cute VLP.

**Table S1.** Bacteria selected from the Tong Lab bacterial library for co-incubation with the samples.

| **Current scientific name** | **Family** | **Number of strains** |
| --- | --- | --- |
| *Pseudomonas aeruginosa* | *Pseudomonadaceae* | 41 |
| *Serratia marcescens* | *Yersiniaceae* | 1 |
| *Enterobacter cloacae* | *Enterobacteriaceae* | 1 |
| *Aeromonas hydrophila* | *Aeromonadaceae* | 1 |
| *Enterococcus faecium* | *Enterococcaceae* | 2 |
| *Staphylococcus haemolyticus* | *Staphylococcaceae* | 6 |
| *Staphylococcus hominis* | *Staphylococcaceae* | 1 |
| *Proteus mirabilis* | *Morganellaceae* | 1 |
| *Proteus penneri* | *Morganellaceae* | 1 |
| *Achromobacter xylosoxidans* | *Alcaligenaceae* | 1 |
| *Morganella morganii* | *Morganellaceae* | 1 |
| *Bacillus cereus* | *Bacillaceae* | 1 |
| *Staphylococcus aureus* | *Staphylococcaceae* | 10 |
| *Citrobacter freundii* | *Enterobacteriaceae* | 3 |
| *Klebsiella pneumoniae* | *Enterobacteriaceae* | 13 |
| *Escherichia coli* | *Enterobacteriaceae* | 10 |
| *Staphylococcus epidermidis* | *Staphylococcaceae* | 4 |
| *Acinetobacter baumannii* | *Moraxellaceae* | 17 |

**Table S2.** NCBI GenBank number of the replicase reference sequence.

| **Description** | **GenBank accession no.** |
| --- | --- |
| Klebsiella phage Cute | OP321095 |
| Escherichia phage Qbeta | NC_001890.1 |
| Enterobacteria phage M11 | AF052431.1 |
| Enterobacteria phage SP | GQ153932.1 |
| Bacteriophage NL95 | AF059243.1 |
| Enterobacteria phage FrBurgundy | PP430142.1 |
| Enterobacteria phage FrHibiscus | PP430143.1 |
| Enterobacteria phage FrMerlot | PP430144.1 |
| Enterobacteria phage FrSangria | PP430145.1 |
| Escherichia phage MS2 | MK213795.1 |
| Enterobacteria phage GA | NC_001426.1 |
| Enterobacteria phage fr | X15031.1 |
| Enterobacteria phage M12 | AF195778.1 |
| Enterobacteria phage FrBlood | PP430139.1 |
| Enterobacteria phage FrHenna | PP430140.1 |
| Pseudomonas phage PP7 | NC_001628.1 |
| ssRNA phage Gerhypos.1_9 | NC_073906.1 |
| ssRNA phage Gerhypos.3_17 | NC_073907.1 |
| ssRNA phage SRR5208570_1 | NC_073909.1 |

**Table S3.** qPCR primer sequence of the phage Cute *A1* gene.

| **Gene name** | **Primer direction** | **Sequence** |
| --- | --- | --- |
| *A1* | Primer FOR | CTAAGCATGAGGAACGACTATC |
| *A1* | Primer REV | TTTGGCGCGATCCATCTA |
